# Oral and fecal microbiota perturbance in cocaine users: Can rTMS-induced cocaine abstinence support eubiosis restoration?

**DOI:** 10.1016/j.isci.2023.106627

**Published:** 2023-04-20

**Authors:** Elisabetta Gerace, Simone Baldi, Maya Salimova, Leandro Di Gloria, Lavinia Curini, Virginia Cimino, Giulia Nannini, Edda Russo, Marco Pallecchi, Matteo Ramazzotti, Gianluca Bartolucci, Brunella Occupati, Cecilia Lanzi, Maenia Scarpino, Giovanni Lanzo, Antonello Grippo, Francesco Lolli, Guido Mannaioni, Amedeo Amedei

**Affiliations:** 1 Department of Neuroscience, Psychology, Drug Research and Child Health NEUROFARBA, University of Florence, 50139 Florence, Italy; 2 Department of Health Sciences, Clinical Pharmacology and Oncology Unit, University of Florence, 50139 Florence, Italy; 3 Department of Experimental and Clinical Medicine, University of Florence, 50134 Florence, Italy; 4 Azienda Ospedaliera Universitaria di Careggi, Clinical Toxicology and Poison Control Centre, 50134 Florence, Italy; 5 Department of Biomedical, Experimental and Clinical Sciences “Mario Serio”, University of Florence, 50134 Florence, Italy; 6 Azienda Ospedaliera Universitaria di Careggi, Neurophysiology Unit, 50134 Florence, Italy; 7 Interdisciplinary Internal Medicine Unit, Careggi University Hospital, 50134 Florence, Italy

**Keywords:** Substance of abuse, Microbiome

## Abstract

The effects of cocaine on microbiota have been scarcely explored. Here, we investigated the gut (GM) and oral (OM) microbiota composition of cocaine use disorder (CUD) patients and the effects of repetitive transcranial magnetic stimulation (rTMS). 16S rRNA sequencing was used to characterize GM and OM, whereas PICRUST2 assessed functional changes in microbial communities, and gas-chromatography was used to evaluate fecal short and medium chain fatty acids. CUD patients reported a significant decrease in alpha diversity and modification of the abundances of several taxa in both GM and OM. Furthermore, many predicted metabolic pathways were differentially expressed in CUD patients’ stool and saliva samples, as well as reduced levels of butyric acid that appear restored to normal amounts after rTMS treatment. In conclusion, CUD patients showed a profound dysbiotic fecal and oral microbiota composition and function and rTMS-induced cocaine abstinence determined the restoration of eubiotic microbiota.

## Highlights

• The CUD patients showed a marked microbial (fecal and oral) dysbiosis
• We documented lower fecal levels of butyric acid on CUD patients
• The rTMS treatment partly restored fecal butyric acid levels in CUD patients
• CUD patients significantly showed different microbial metabolic functions than controls

## Graphical abstract

**Figure:**
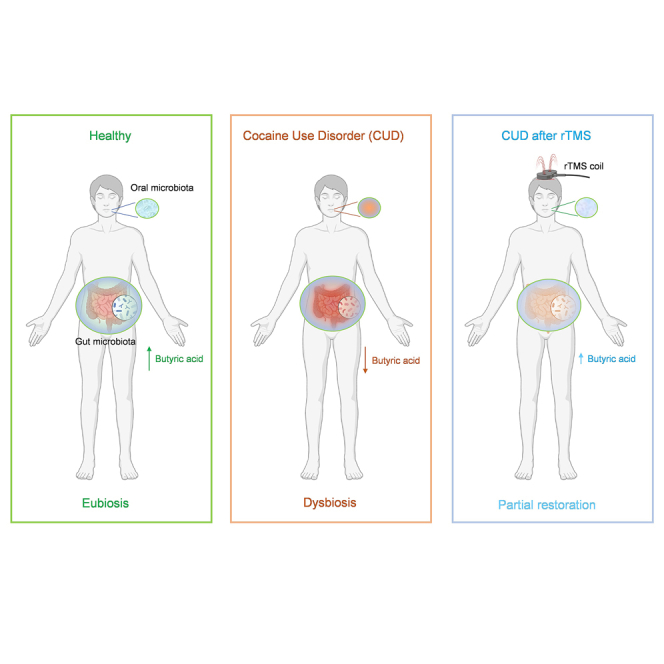

## Introduction

Microbiota dysfunction has been associated with several diseases and a significant body of evidence has identified microbial dysbiosis as a contributor to cognitive and neuropsychiatric disorders.^1^^,^^2^ Recent findings elucidated the importance of the gut microbiota (GM) and its metabolites, especially short chain fatty acids (SCFAs), in the bidirectional communication between the central and enteric nervous systems, named the gut-brain axis.^3^^,^^4^ Hence, an alteration of this complex crosstalk, that involve sensory, endocrine, and immune signals, has been suggested to play a prominent role in the pathophysiology of different unhealthy conditions, including substance use disorders (SUD).^5^

The relationship between GM dysbiosis and SUD has been widely reported in rodents^6^^,^^7^^,^^8^ and humans.^9^^,^^10^^,^^11^ In addition, an increased intestinal barrier permeability (named leaky gut), and consequently, an altered gut-brain communication has been observed in SUD, especially in alcohol use disorders (AUD).^12^^,^^13^ Indeed, alcohol-induced dysbiosis has been the subject of numerous review articles^13^^,^^14^^,^^15^^,^^16^^,^^17^ and Li et al. have interestingly documented that the GM transfer from AUD patients to germ-free animals caused learning and memory dysfunctions, depression and anxious behaviors, suggesting the pivotal role of GM in AUD development.^18^

Many drugs of abuse have also been reported to have a detrimental effect on the composition and function of GM.^11^ For instance, several studies have associated opiates, in particular their chronic use, with substantial alterations in the GM of humans^19^^,^^20^ and nonhuman primates.^21^ Among drugs of abuse, also nicotine,^7^^,^^22^^,^^23^ methamphetamine^24^^,^^25^ and cannabinoids^26^^,^^27^ have been reported to cause significant GM dysbiosis.

Among SUD, cocaine use disorder (CUD) currently represents a major public health problem worldwide, especially in Western countries.^28^ Cocaine is a psychoactive and addictive substance that, when used chronically, provokes a dysregulation of neurotransmitter systems (especially dopamine pathways),^29^^,^^30^ harmful immune activation, metabolic derangements and finally gastrointestinal symptoms (e.g., anorexia, nausea, vomiting and diarrhea) that significantly perturb the GM, which in turn compromises the host’s nutritional status.^31^^,^^32^^,^^33^

However, cocaine’s effects on intestinal and oral microbial communities have been poorly investigated to date. Volpe et al. evaluated the GM structure in cocaine users with HIV infection to explore potential alterations in intestinal bacterial composition and, compared to non-users, they reported a higher relative abundance of Bacteroidetes.^34^ Instead, as recently reported by Fu et al., the oral microbiota (OM) of cocaine users showed a decreased salivary microbial diversity compared to non-users. In detail, several genera resulted decreased in saliva samples of cocaine-addicted patients whereas *Streptococcus* spp.was the only genus significantly enriched.^35^ Furthermore, the induction in animal models of a cocaine-mediated gut dysbiosis resulted to be associated either with an upregulation of pro-inflammatory mediators that alters gut-barrier integrity by impairing epithelial permeability or a modification of behavioral responses.^36^^,^^37^

Hence, in recent years, several trials have been performed to identify an adequate and effective treatment for CUD, but all the proposed pharmacological therapies, such as dopamine or γ-aminobutyric acid (GABA) agonists and glutamate antagonists, resulted in elusive and modest long-term success rates.^38^ On the other hand, repetitive transcranial magnetic stimulation (rTMS) has been proven to be an alternative and non-pharmacological therapeutic perspective effective in CUD treatment.^39^^,^^40^^,^^41^^,^^42^ rTMS represents a safe and non-invasive form of brain stimulation that modulates the cellular activity of the cerebral cortex through a magnetic pulse applied to selected brain areas.^43^ Our recent publications, in line with other previously published reports,^39^^,^^40^^,^^41^^,^^42^ documented its positive effect on the reduction of both cocaine intake and craving in CUD patients.^44^^,^^45^

Therefore, the present study aims to evaluate the fecal and oral microbiota composition and function in patients with CUD and to examine whether the positive effects of rTMS treatment on cocaine consumption can also favor the restoration of eubiotic microbiota and pave the way to the future development of supplemental strategies for the management of complications correlated with cocaine abuse.

## Results

### Patients’ enrollment

For the present study, 58 patients with CUD and 20 volunteer healthy controls (HC) (17 males, 3 females; with a mean age of 43.8 ± 7.9) have been enrolled. Clinical patients’ features are reported in Table S1 and, in detail, the mean age at enrollment was 40.4 years (range 20–58 years) and 83% of them were male. Of the recruited CUD patients, 62% reported consuming cocaine for more than ten years and 29% took it one time per day. The cocaine consumption was 84% in a single modality, specifically: 67% sniffing, 37% smoking and 16% intravenous. In addition, enrolled patients confirmed to usually consume alcohol (67%), tobacco (76%), tetrahydrocannabinol (THC; 35%) opioids (5%), methadone (5%) or amphetamine (2%). Unfortunately, a high dropout (nearly 60%) was observed during the treatments both in rTMS (n = 18; 58%) and sham group (n = 16; 57%) but patients completing the study did not differ significantly from those dropping for frequency and pattern of cocaine use.

### Gut and oral microbiota composition

**Figure 1:**
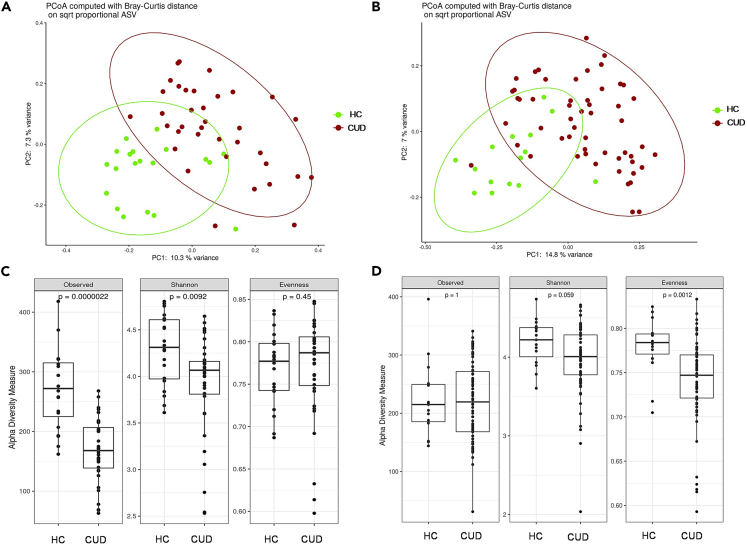
General description of the bacterial communities of HC and CUD patients (A) Principal coordinate analysis (PCoA) conducted with Bray-Curtis dissimilarity of the bacterial communities of fecal samples among HC and CUD patients. (B) PCoA conducted with Bray-Curtis dissimilarity of the bacterial communities of saliva samples among HC and CUD patients. (C) Boxplots showing alpha diversity indices (Observed ASV, Shannon index, Pielou’s evenness) of fecal samples among HC and CUD patients. (D) Boxplots showing alpha diversity indices (Observed ASV, Shannon index, Pielou’s evenness) of saliva samples among HC and CUD patients.

We first evaluated whether CUD patients showed a different intestinal and oral microbiota structure in comparison with HC. Rarefaction curves for observed ASVs revealed that both fecal and saliva specimens were sufficiently sampled (Figure S1) and, in detail, the principal coordinate analysis computed using the Bray-Curtis dissimilarity metric highlighted a clear separation among fecal (PERMANOVA, p< 0,0001) (Figure 1A) and saliva samples (PERMANOVA, p< 0.0001) (Figures 1B) of HC and CUD patients. Statistically significant beta diversity, namely the variability in microbial community composition (the identity of the observed taxa) among samples, between fecal or saliva samples of HC and CUD patients was also found at all taxonomic ranks (Table S2). In addition, as depicted in Figures 1C and 1D, a reduced alpha diversity, which is a measure that summarizes the richness (number of taxonomic groups) and evenness (distribution of abundances of the groups) of a microbial community, was reported in both GM (observed ASV richness, p = 2.2e-6; Shannon index, p = 0.0092) and OM (Shannon index, p = 0.059; Pielou’s evenness, p = 0.001) of CUD patients compared to HC.

**Figure 2:**
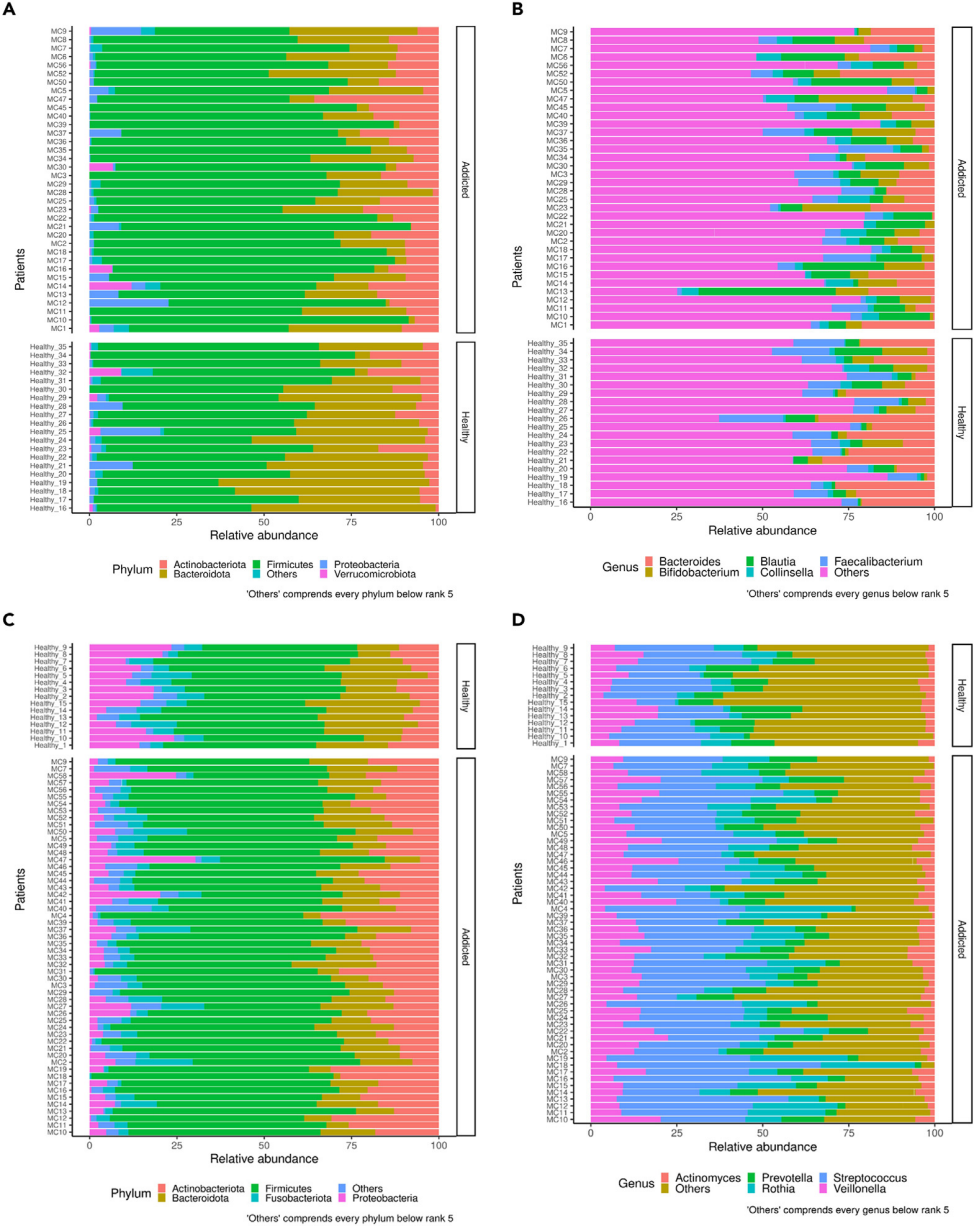
Stacked bar graphs showing relative bacterial abundances at phylum and genus level among HC and CUD patients (A) Stacked bar plots showing the five most abundant phyla in fecal samples of HC and CUD patients. (B) Stacked bar plots showing the five most abundant genera in fecal samples of HC and CUD patients. (C) Stacked bar plots showing the five most abundant phyla in saliva samples of HC and CUD patients. (D) Stacked bar plots showing the five most abundant genera in saliva samples of HC and CUD patients.

Taxonomic analysis of fecal and saliva samples is detailed in Table S3 and the stacked bar-plot representation displayed a marked different relative abundance of both the top five phyla and genera in either fecal (respectively reported in Figures 2A and 2B) and saliva (respectively reported in Figures 2C and 2D) samples collected from CUD patients and HC. In detail, the top five phyla in stool samples were Actinobacteriota, Bacteroidota, Firmicutes, Proteobacteria, and Verrucomicrobiota whereas saliva samples showed high abundances of Actinobacteriota, Bacteroidota, Firmicutes, Fusobacteria, and Proteobacteria. Besides, the top five genera in stool samples were *Bacteroides*, *Bifidobacterium*, *Blautia*, *Collinsella*, and *Faecalibacterium* whereas saliva samples showed high abundances of *Actinomyces*, *Prevotella, Rothia*, *Streptococcus* and *Veillonella*.

**Figure 3:**
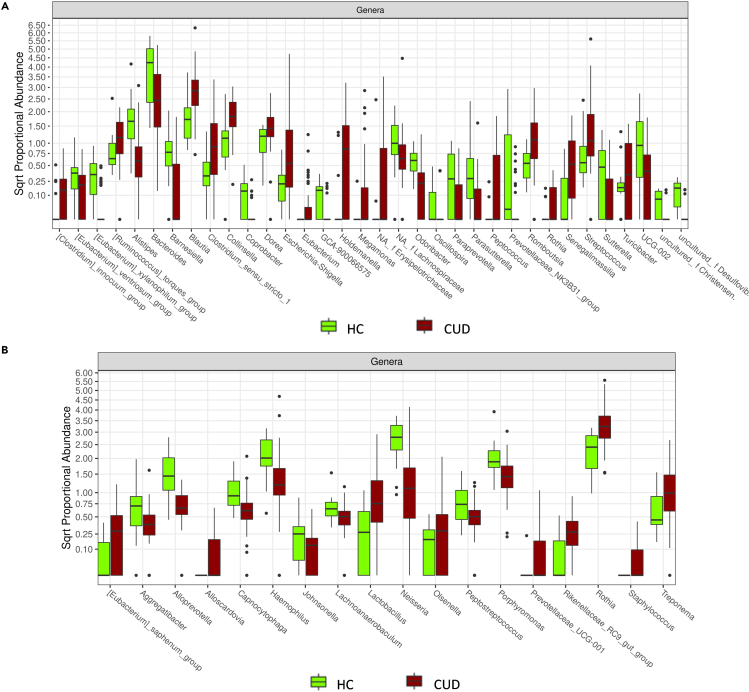
Representation of the significant differentially abundant taxa between HC and CUD patients (A) Boxplots reporting the significant differentially abundant taxa among fecal samples of HC and CUD patients. (B) Boxplots reporting the significant differentially abundant taxa among saliva samples of HC and CUD patients. All the presented results have an adj. p< 0.05.

Subsequently, differential abundance analyses were performed at all taxonomic ranks and notably several taxa resulted differentially abundant in fecal (Figure 3A, Table S4) and saliva (Figure 3B, Table S5) samples of HC and CUD patients. In detail, compared to HC, CUD patients reported higher fecal abundances of Erysipelotrichaceae, *Blautia spp.*, *Collinsella spp.*, *Dorea spp.*, *Romboutsia spp.* and *Streptococcus spp*. but reduced levels of Christensenellaceae, Desulfovibrionaceae, Lachnospiraceae, *Alistipes spp.*, *Bacteroides spp*.*,* and *Barnesiella spp.* On the other hand, CUD patients showed higher saliva levels of *Rothia spp.*, *Staphylococcus spp.* and *Treponema* spp. but lower levels of *Haemophilus* spp., *Neisseria spp.* and *Porphyromonas spp.* than HC.

### Functional analysis of the fecal and oral microbiota

The PICRUSt2 (phylogenetic investigation of communities by reconstruction of unobserved states) predictive metabolism approach was used on the 16S rRNA gene sequencing data to assess a functional analysis of the GM of CUD patients and HC. As shown in Figure S2A, based on the MetaCyc metabolic pathway database (www.metacyc.org), we found 124 significant enriched pathways (LDA>2.0) in fecal samples; of which 63 resulted more abundant in CUD patients whereas 61 resulted more expressed in HC. Of interest, CUD patients showed an up-regulated profile in purine degradation (super pathway of purine deoxyribonucleosides degradation, p= 0.002; purine nucleobases degradation I, p= 0.001) and in pyrimidine biosynthesis (pyrimidine deoxyribonucleotides *de novo* biosynthesis IV, p= 0.001; pyrimidine deoxyribonucleotides biosynthesis from CTP, p= 0.001). Moreover, the pathways implicated in amino acids biosynthesis, such as arginine (L-arginine biosynthesis I (via L-ornithine), p= 0.011; L-arginine biosynthesis IV (archaea), p= 0.015), tyrosine (super pathway of L-tyrosine biosynthesis, p= 0.008), aspartate (aspartate super pathway, p= 0.007), glutamate and glutamine (L-glutamate and L-glutamine biosynthesis, p= 0.003) and lysine, threonine and methionine (super pathway of L-lysine, L-threonine and L-methionine biosynthesis p= 2.06e5) resulted more expressed in CUD patients than in HC. Furthermore, the super pathway of phospholipid biosynthesis (p= 0.011), crucial in promoting inflammation via LPS production and the pentose phosphate pathway (p= 0.003), were both higher in CUD patients than in HC. On the other hand, HC showed a higher expression of pathways related to purine biosynthesis (super pathway of purine nucleotides *de novo* biosynthesis I, p= 0.004; super pathway of purine nucleotides *de novo* biosynthesis II, p= 0.005).

Regarding the saliva samples (Figure S2B), we found 149 significant enriched pathways (LDA>2.0) and CUD patients showed an up-regulated profile in isoleucine (L-isoleucine biosynthesis I (from threonine), p = 0.001; L-isoleucine biosynthesis II, p = 0.004; L-isoleucine biosynthesis III, p = 0.001) and valine (L-valine biosynthesis, p = 0.001) synthesis and also in both purine and pyrimidine biosynthesis (adenine and adenosine salvage III, p = 0.001; super pathway of adenosine nucleotides *de novo* biosynthesis II, p = 0.001) and degradation (purine ribonucleosides degradation, p = 7.56e4; super pathway of pyrimidine deoxyribonucleosides degradation, p = 1,37e5).

### Fecal SCFAs and MCFAs profile

**Figure 4:**
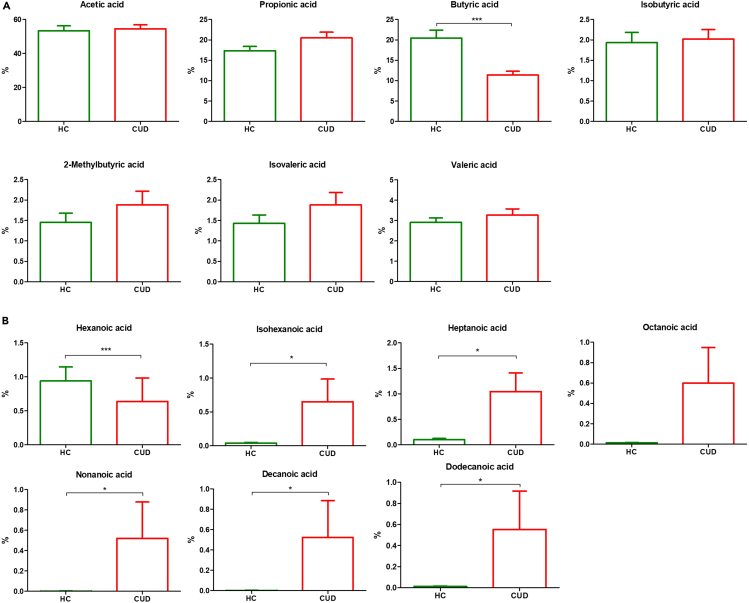
Representation of fecal SCFAs and MCFAs abundances between HC and CUD patients (A) Bar plots representing the fecal SCFAs abundances between HC and CUD patients. (B) Bar plots representing the fecal MCFAs abundances between HC and CUD patients. Analyses were assessed using the Mann-Whitney test and p-values less than 0.05 were considered statistically significant. The asterisks (∗) represent p-values, ∗p < 0.05, ∗∗p < 0.01, ∗∗∗p < 0.001.

We used a GC-MS method to evaluate the microbial-derived SCFAs (acetic, propionic, butyric, isobutyric, isovaleric 2-methylbutyric and valeric acids) and MCFAs (hexanoic, isohexanoic, heptanoic, octanoic, nonanoic, decanoic and dodecanoic acids) abundances in fecal samples of HC and CUD patients. Because these analyses could be in part influenced by the total amount of each metabolite, we performed the comparisons on the SCFAs and MCFAs percentage compositions (Table S6). Regarding SCFAs, as displayed in Figure 4A, we found that CUD patients reported a significantly (p<0.001) lower level of butyric acid than HC. On the other hand, compared to HC, CUD patients showed a lower level of hexanoic acid (p<0.001) but higher abundances of isohexanoic (0.014), heptanoic (p= 0.028), nonanoic (p= 0.019), decanoic (p= 0.015) and dodecanoic acids (p= 0.019) (Figure 4B).

### Effect of rTMS treatment

**Figure 5:**
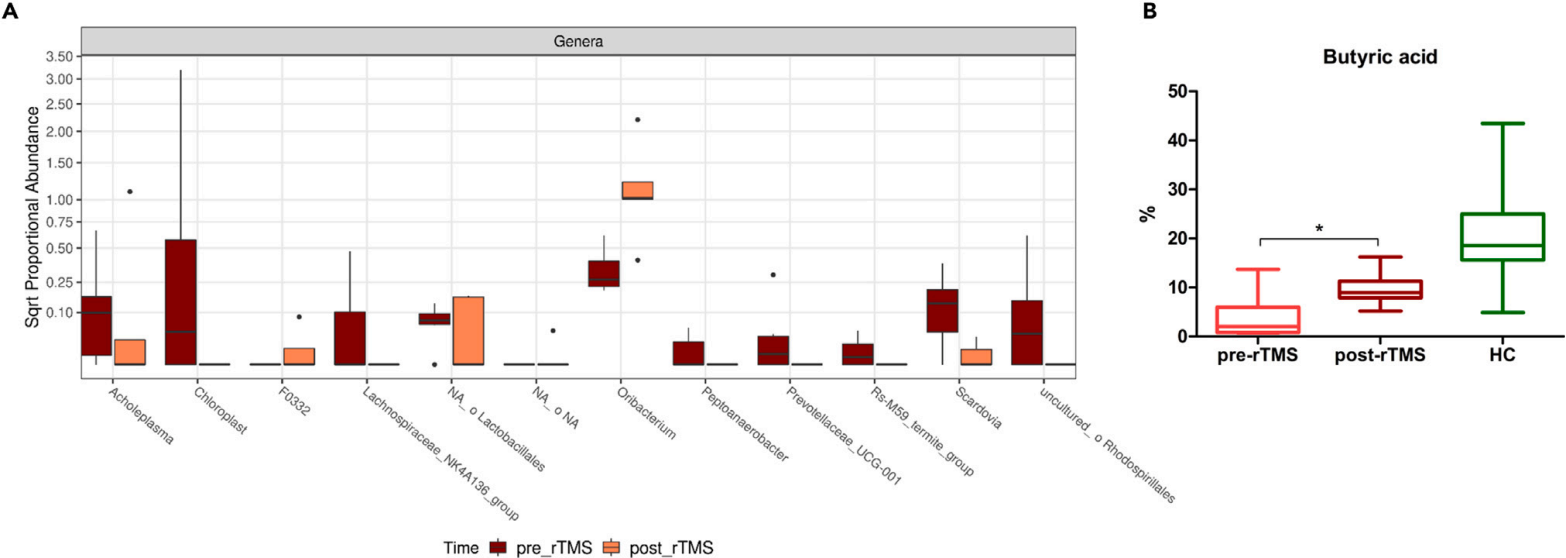
rTMS effects on CUD patients (A) Boxplots reporting the salivary significant differentially abundant taxa in CUD patients pre- and post-rTMS intervention. All the presented results have an adj. p< 0.05. (B) Boxplot reporting the statistically significant increase of fecal butyric acid in CUD patients after rTMS intervention. Analyses were assessed using the paired Wilcoxon signed-rank test and p-values less than 0.05 were considered statistically significant. The asterisks (∗) represent p-values, ∗p < 0.05, ∗∗p < 0.01, ∗∗∗p < 0.001.

Considering the beneficial effect of rTMS in reducing cocaine cravings, we evaluated whether it also determined compositional or functional modifications in both GM and OM of CUD patients 8 weeks after rTMS treatment. As shown in Figure 5A, in patients who underwent active rTMS treatment a significant reduction of the order Lactobacillales (log2FC= 7,076; adj. p= 0.043) and of *Acholeplasma spp.* (log2FC= 7,522; adj. p= 0.032), *Chloroplast spp.* (log2FC= 35,572; adj. p= 1,02e-19), Lachnospiraceae*_NK4A136_group spp.* (log2FC= 21,703; adj. p= 1,36e-07), *Peptoanaerobacter spp.* (log2FC= 23,236; adj. p= 1,29e-08), Prevotellaceae*_UCG-001 spp.* (log2FC= 21,250; adj. p= 2,43e-11), *Rs-M59_termite_group* (log2FC= 27,708; adj. p= 4,92e-12) and *Scardovia spp.* (log2FC= 5,374; adj. p= 0.002) was observed in saliva samples. Instead, a significant increase of *F0332 spp.* (log2FC= −32,659; adj. p= 1,47e-16) and *Oribacterium spp.* (log2FC= −1,588; adj. p= 0.038) have been reported in OM of CUD patients. Furthermore, although no statistically significant taxa have been found in GM of CUD patients after rTMS treatment, a significant increase in the fecal abundance of butyric acid in post-rTMS samples has been reported (Figure 5B).

## Discussion

To date, much of the research on cocaine addiction has been focused on the neurological and genetic aspects of consumer behavior. However, recent preclinical studies of intestinal dysbiosis in animal models and people with cocaine addiction have strengthened the presence of bidirectional communication between the enteric and the central nervous system.^46^ In the present study, we have evaluated, for the first time in humans, the fecal and oral microbiota composition and functionality in patients affected by CUD. In addition, we examined whether the positive effects of rTMS treatment on cocaine consumption can also support the restoration of eubiotic microbiota.

Our results showed that both oral and intestinal microbiota structures present a significant reduction of the alpha diversity between CUD patients and HC with a significant parting between CUD patients and HC fecal and saliva samples. These data are in line with Scorza et al. who documented a reduced alpha diversity in the GM of cocaine-treated rats compared to the control group.^47^ Moreover, Fu and collaborators found a significantly lower alpha diversity in the OM of cocaine users compared to non-users.^35^

In particular, the differential analysis at all taxonomic ranks performed in fecal from CUD patients reveals significantly increased abundances of Erysipelotrichaceae, *[Clostridium]_innocuum_group spp.*,*[Ruminococcus]_torques_group spp.*, *Blautia spp.*, *Clostridium_sensu_stricto spp.*, *Collinsella spp.*, *Dorea. spp.*, *Escherichia-Shigella spp.*, *Eubacterium spp.*, *Holdemanella spp.*, *Megamonas spp.*, *Peptococcus spp.*, *Romboutsia spp.*, *Rothia spp.*, *Senegalimassilia spp.*, *Streptococcus spp.*, *Turicibacter spp.* and reduced levels of Christensenellaceae, Desulfovibrionaceae, Lachnospiraceae, *[Eubacterium]_ventriosum_group spp.*, *[Eubacterium]_xylanophilum_group spp.*, *Alistipes. spp.*, *Bacteroides. spp.*, *Barnesiella spp.*, *Coprobacter spp.*, *GCA-900066575 spp.*, *Odoribacter spp.*, *Oscillospira spp.*, *Paraprevotella spp.*, *Parasutterella spp.*, Prevotellaceae*_NK3B31_group spp.*, *Sutterella spp.* and *UCG-002 spp*.

Our findings are supported by preclinical studies in mice, where Erysipelotrichaceae members and *Turicibacter* *spp.* were increased in fecal droppings of cocaine-exposed mice compared to controls.^37^ Besides, cocaine administration depletes the Lachnospiraceae members in the rodent gut^47^ and both *Paraprevotella* and *Parasutterella* species were decreased in long-term SUD subjects.^48^ Of interest, the association between *Clostridium innocuum* and neuroticism, a symptom of cocaine-induced psychosis, has been recently documented in cocaine-dependent patients.^49^^,^^50^ However, in contrast to our findings, the GM of cocaine-exposed mice showed increased abundances of Desulfovibrionaceae*, Alistipes. spp. Barnesiella spp.*, *Odoribacter. spp.* and *Streptococcus spp.*^37^^,^^51^ Remarkably, also human alcohol over-consumers displayed increased intestinal levels of pro-inflammatory *Sutterella spp.*, suggesting that substance abuse led to an increased inflammatory tone.^52^ Analyzing the OM of CUD patients, we observed that cocaine abuse determined the salivary increase of [*Eubacterium]_saphenum_group spp.*, *Alloscardovia spp.*, *Lactobacillus spp.*, *Olsenella spp.,* Prevotellaceae*_UCG-001 spp.*, *Rikenellaceae_RC9 spp.*, *Rothia spp.* and *Treponema.spp.* and the reduction of the genera *Aggregatibacter*, *Alloprevotella*, *Capnocytophaga*, *Haemophilus*, *Johnsonella*, *Lachnoanaerobaculum*, *Neisseria*, *Peptostreptococcus*, and *Porphyromonas*.

In support of our findings, cocaine addiction has been associated with reduced abundances of *Haemopilus.spp.*, *Neisseria. spp.* and *Porphyromonas spp.*^35^ as well as users of other substance abuse showed higher levels of Prevotellaceae and *Lactobacillus. spp*. than controls.^53^^,^^54^ Notably, *Lachnoanaerobaculum spp.* and *Capnocytophaga spp*. resulted respectively increased and reduced also in alcohol-addicted patients,^55^ whereas abstinence from alcohol has been associated with *Peptostreptococcus spp* depletion.^56^ In addition, our results displayed a significant increase of *Treponema* *spp., Staphylococcus spp., Rothia spp.* and *Olsenella spp.* in CUD patients, all genera related to periodontal inflammation and other oral diseases.^57^^,^^58^^,^^59^^,^^60^

In general, these microbial compositional alterations in both OM and GM of CUD patients reflect the presence of a remarkably dysbiotic condition. In fact, although a eubiotic intestinal or buccal environment is constituted by a high richness of species,^61^ the reduction of the fecal or saliva microbial diversity and the increase in pro-inflammatory species have been widely reported as a reflection of intestinal or oral dysbiosis.^10^^,^^62^

Therefore, to better understand the consequence of all these changes in GM and OM, we performed the predictive functional analysis using the PICRUSt2 software. We observed that various metabolic functions of either salivary or intestinal microbiota resulted differentially expressed between CUD patients and HC. We found that CUD patients showed higher amino acids and glutamate biosynthesis in the GM, compared to HC and, accordingly, Blanco et al. reported that brain glutaminase expression and activity can be modulated by cocaine in mice.^63^ Moreover, the alteration of the tyrosine ratio has also been associated with cocaine and ethanol abuse.^64^

The several enhanced inflammatory pathways found in CUD patients confirmed that the inflammatory tone in chronic cocaine consumers has been regarded as consequential to behaviors that occur in conjunction with cocaine-related changes in brain function.^65^ For instance, the microbial dysbiosis caused by drug consumption determines the degradation of the intestinal epithelium which is responsible for the consequent translocation of either microbe or microbial-derived metabolites that alter the bidirectional communication throughout the gut and brain.^66^ For example, microbial products, such as neuromodulators (i.e., dopamine, serotonin, GABA) or SCFAs, which can diffuse through the epithelial walls, can directly act on the central nervous system through the vagus nerve or influence the brain physiology by passing the blood-brain barrier and exerting various effects.^67^^,^^68^ For instance, several effects may be obtained in the induction of antidepressant-like responses and modulation of serotoninergic, GABAergic and dopaminergic neurotransmissions, all process determinants for the modulation of crucial areas for reward behaviors like the hippocampus and striatum.^69^^,^^70^^,^^71^^,^^72^ Of interest, preliminary evidence also demonstrated that cocaine-induced perturbations of GM are associated with different reward responses, suggesting the role of GM dysbiosis in driving drug-seeking behavior.^73^

Parallel to the ascertained gut-brain interplay, in the last years has been documented that also oral resident microbes in the mouth can communicate with the brain through specific mechanisms such as (1) microbial and metabolite escape, (2) modulation of neuroinflammation, (3) modulation of brain signaling and (4) response to neurohormones.^74^ In particular, Narengaowa et al. recently documented that OM dysbiosis promotes the development of Aβ plaques and neurofibrillary tangles in Alzheimer’s disease patients, hypothesizing a direct interaction of oral bacterial species and their products through the trigeminal/olfactory/facial nervous system.^75^

About this, our results show that pathways involved in isoleucine and valine biosynthesis were highly expressed by the salivary microbiota of CUD patients compared to HC. Both isoleucine and valine are included in the group of branched-chain amino acids (BCAAs) that serve as substrates for protein synthesis or energy production and perform several metabolic and signaling functions. Notably, they are transported into the brain via the same carrier that transports phenylalanine, tyrosine and tryptophan, making competition that may influence the synthesis of some neurotransmitters, especially dopamine, norepinephrine, and serotonin.^76^^,^^77^ Therefore, their increase may influence neurotransmitter levels in the brain with effects on its function and behavior, that may occur in CUD patients. Indeed, high concentrations of BCAAs were found to be neurotoxic because of increased excitotoxicity and oxidative stress.^78^^,^^79^ Moreover, in CUD patients we reported increased expression of pentose phosphate pathways and pyruvate fermentation, which are both implicated in the modulation of both chronic neuroinflammation and dopaminergic neurodegeneration as well as associated with multiple sclerosis pathogenesis.^80^^,^^81^

The evaluation of fecal SCFAs and MCFAs also confirmed a functional GM alteration, in fact, CUD patients showed lower levels of butyric and hexanoic acids but higher abundances of isohexanoic, heptanoic, nonanoic, decanoic and dodecanoic acids.

The SCFAs, especially acetic, propionic and butyric acids, are the main end-products of the bacterial fermentation of dietary fibers that exert crucial immunomodulatory and physiological effects on several organs including the brain. In particular, butyric acid is an attractive therapeutic molecule because of its wide array of biological functions, such as its ability to serve as a histone deacetylase (HDAC) inhibitor, an energy metabolite to produce ATP and a G protein-coupled receptor (GPCR) activator and, pharmacologically, butyrate has a profoundly beneficial effect on neurodegenerative and psychological disorders.^82^ As reported by Chivero et al., cocaine consumption specifically depletes key SCFA producers’ abundances.^37^^,^^83^

Instead, although MCFAs can be metabolized to ketones by astrocytes to be used as an energy source for the brain^84^ they display remarkable pro-inflammatory effects by enhancing the production of pro-inflammatory cytokines and reducing the anti-inflammatory IL-10 levels through TLR2 activation.^85^ Moreover, a recent *in vivo* study has documented that free fatty acid receptor 1, which is a G protein-coupled receptor for MCFAs, has a facilitatory role in striatal serotonin release, with a consequent enhancement of cocaine-induced locomotor activity.^86^

Finally, considering our previously reported beneficial effect of rTMS treatment in lowering cocaine craving and consumption^45^ and taking into account the aforementioned interplay between the brain and both oral and intestinal microbiota, we have also evaluated the compositional and functional modifications in both GM and OM of CUD patients after 8 weeks rTMS treatment. Although no significant differences have been found in GM composition, rTMS-subjects reported a significant increase in fecal butyric acid abundance, an SCFA with potent anti-inflammatory effects in the gut and an important neuroprotective role in the brain.^87^^,^^88^ CUD patients exposed to rTMS also showed in the OM a significant increase of *F0332 spp.* and *Oribacterium. spp.* and the reduction of Lactobacillales, *Acholeplasma spp.*, *Chloroplast. spp.,* Lachnospiraceae*_ NK4A136_group spp.*, *Peptoanaerobacter. spp.*, Prevotellaceae*_UCG-001 spp.*, *Rs-M59_termite_group*, and *Scardovia spp.* Although increased levels of *Peptoanaerobacter stomatis* and *Scardovia wiggsiae* have been associated with periodontal disease,^89^^,^^90^ rTMs treatment has also determined the beneficial reduction of Lactobacillales because drug users reported an increase in *Lactobacillus spp.*^54^ Moreover, *Oribacterium* species resulted to be more abundant in healthy individuals^91^ whereas *Acholeplasma spp.* was abundant in patients with periodontal disease.^92^ Hence, this emerging therapeutic strategy not only showed positive neuromodulation effects in CUD but also results in a beneficial compositional OM modification and a change in GM function through neurobiological mechanisms not yet fully understood. However, the effects of rTMS in inducing changes in neurotransmitter systems have been largely demonstrated, specifically in the alterations of striatal dopamine release and metabolite levels, as well as to glutamate transporter and receptor expression, which may be relevant to improving the aberrant plasticity observed in individuals with SUD.^93^

Moreover, it is well known that CUD is strictly associated with depression and the efficacy of rTMS in the treatment of depression has been demonstrated by many studies. Through the modulation of the hypothalamic-pituitary-adrenocortical (HPA) axis, rTMS can directly or indirectly prevent hippocampal neuron atrophy and apoptosis and alleviate the symptoms of depression.^94^ In addition, Ferrulli et al. have recently associated an rTMS rebalancing of the intestinal microbiota with norepinephrine variations.^95^ In fact, rTMS has been suggested to induce a norepinephrine transporter upregulation, with consequent central and gut luminal norepinephrine levels, with consequent beneficial effects on GM composition.

Thus, we can speculate that rTMS-induced cocaine abstinence could play a role in the gut-brain axis and exert effects on microbial communities leading to GM and OM beneficial properties.

Furthermore, the remarkable alteration of both oral and intestinal microbiota structure and function in CUD patients confirmed the evidence suggesting the important role of microbes in the pathogenesis of neuropsychiatric pathologies, including reward processes and substance-related disorders.^96^^,^^97^

To conclude, our study demonstrates the profound oral and intestinal compositional and functional dysbiosis of CUD patients and that rTMS treatment inducing reduced cocaine consumption and craving may represent a promising avenue for future therapeutic development.

### Limitations of the study

Although we have analyzed for the first time both GM and OM composition and function in CUD patients and the effects of rTMS, we are aware that this study has some limitations such as the enrollment of CUD patients that also consumes other drugs including alcohol (e.g., opiates, nicotine, methamphetamine, cannabinoids) which can influence fecal and buccal microbiota composition and function.^11^^,^^23^^,^^24^^,^^26^ Moreover, other limitations of this study are represented by the restricted number of enrolled patients with an understandable high dropout, the low taxonomical resolution of 16S rRNA sequencing, and the not consideration of the possible unbalanced dietary habits of CUD patients. Despite this, we have documented various and consistent differences in the gut and oral microbial communities of CUD patients that are often sustained by several animal studies and the only two human studies present in the literature. Importantly, our work suggests the OM relevance as a future investigative tool for several diseases, including SUD, and indicates that a diet rich in butyrate could be used as a therapy to treat CUD preventing some of the frequent relapses by improving their cognitive abilities.

## STAR★Methods

### Key resources table

**Table:** 

REAGENT or RESOURCE	SOURCE	IDENTIFIER
**Biological samples**		

Healthy subjects’ saliva and stool samples	Voluntary donation	This paper
Saliva and stool samples from patient with cocaine use disorder, pre- and post- rTMS intervention	Careggi University Hospital (Florence, Italy) or primary care physicians/Substance Abuse Services in the Florence metropolitan area (Italy)	This paper

**Chemicals, peptides, and recombinant proteins**		

Methanol (Chromasolv grade)	Sigma-Aldrich	Cat# 134860
*tert*-Butyl methyl ether (Chromasolv grade)	Sigma-Aldrich	Cat# 34875
Sodium bicarbonate (Reagent grade)	Sigma-Aldrich	Cat# S6014
Sodium chloride (Reagent grade)	Sigma-Aldrich	Cat# S9888
Hydrochloric acid (Reagent grade)	Sigma-Aldrich	Cat# 320331
[^2^H_3_]Acetic acid (used as internal standard)	Sigma-Aldrich	Cat# 233315
[^2^H_5_]Propionic acid (used as internal standard)	Sigma-Aldrich	Cat# 596507
[^2^H_7_]iso-Butyric acid (used as internal standard)	Sigma-Aldrich	Cat# 632007
[^2^H_9_]iso-Valeric acid (used as internal standard)	Sigma-Aldrich	Cat# 808997
[^2^H_9_]Valeric Acid (used as internal standard)	Sigma-Aldrich	Cat# 493201
[^2^H_11_]Hexanoic Acid (used as internal standard)	Sigma-Aldrich	Cat# 448168
[^2^H_15_]Octanoic Acid (used as internal standard)	Sigma-Aldrich	Cat# 590967
Acetic acid (analytical standards grade)	Sigma-Aldrich	Cat# 71251
Propionic acid (analytical standards grade)	Sigma-Aldrich	Cat# 94425
Butyric acid (analytical standards grade)	Sigma-Aldrich	Cat# 19215
Iso-butyric acid (analytical standards grade)	Sigma-Aldrich	Cat# 46935-U
Valeric acid (analytical standards grade)	Sigma-Aldrich	Cat# 75054
Iso-valeric acid (analytical standards grade)	Sigma-Aldrich	Cat# 78651
2-Methylbutyric acid(analytical standards grade)	Sigma-Aldrich	Cat# 49659
Hexanoic acid (analytical standards grade)	Sigma-Aldrich	Cat# 21529
Iso-hexanoic acid (analytical standards grade)	Sigma-Aldrich	Cat# 277827
Heptanoic acid (analytical standards grade)	Sigma-Aldrich	Cat# 43858
Octanoic acid (analytical standards grade)	Sigma-Aldrich	Cat# 21639
Nonanoic acid (analytical standards grade)	Sigma-Aldrich	Cat# 73982
Decanoic acid (analytical standards grade)	Sigma-Aldrich	Cat# 21409
Dodecanoic acid (analytical standards grade)	Sigma-Aldrich	Cat# 61609

**Critical commercial assays**		

DNeasy PowerSoil ProKit	Qiagen	Cat# 47014

**Deposited data**		

16S rRNA amplicon sequences from saliva and stool samples of 20 healthy volunteers and from saliva and stool samples of 58 patients with cocaine use disorder, pre- and post- rTMS intervention	This paper	Data available in the NCBI Gene Expression Omnibus (GEO) repository, accession number GSE206765

**Software and algorithms**		

R	R Core Team, 2013	v. 4.1
QIIME2	Bolyen et al. (2019)^98^	v. 2021.4
Cutadapt	Martin et al. (2011)^99^	v. 3.4
DADA2	Callahan et al. (2016)^100^	https://benjjneb.github.io/dada2/
PICRUST2	Douglas et al. (2020)^101^	https://huttenhower.sph.harvard.edu/picrust/
phyloseq R package	McMurdie and Holmes (2013)^102^	1.36.0
DESeq2 R package	Love et al. (2014)^103^	1.32.0
vegan R package	Dixon (2003)^104^	2.5-7
ggplot2 R package	Wickham (2016)^105^	3.3.5
dendextend R package	Galili (2015)^106^	1.15.1
LefSe tool	Segata et al. (2011)^107^	https://huttenhower.sph.harvard.edu/lefse/
GraphPad Prism	https://www.graphpad.com/	v.9

**Other**		

TissueLyser LT	Qiagen	Cat# 85600
NanoDrop Spectrofotometer	Thermo Fisher Scientific	Cat# ND-8000-GL
Qubit Fluorometer	Thermo Fisher Scientific	Cat# Q33238
Gas chromatography-mass spectrometry system composed with 5971 single quadrupole mass spectrometer, 5890 gas-chromatograph and 7673 autosampler	Agilent Technologies	This paper
MagPro X100 stimulator	MagVenture	https://www.magventure.com/us/tms-research/products-overview/research-stimulators/stimulators/magpro-x100-4
Generation of amplicons of the variable V3–V4 region of the bacterial 16S rRNA gene and paired-end (2 × 300 cycles) sequencing on the Illumina MiSeq platform	This paper	https://igatechnology.com/
Scripts used for the sequence data analysis	This paper	htps://github.com/LeandroD94/Papers/tree/main/2023_Cocaine_addicted_microbiota_rTMS

### Resource availability

#### Lead contact

Further information and any related requests should be directed to and will be fulfilled by the lead contact, Prof. Amedeo Amedei (amedeo.amedei@unifi.it).

#### Materials availability

This study did not generate new unique reagents.

### Experimental model and subject details

The study involved the Toxicology, Neurophysiology and Psychiatry Units of Careggi University Hospital (Florence, Italy), the Departments of Neuroscience, Psychology, Drug Research and Child Health (NEUROFARBA) and the Department of Experimental and Clinical Medicine of the University of Florence. Patients with CUD diagnosis were recruited according to the Diagnostic and Statistical Manual of Mental Disorders, Fifth Edition, (DSM-IV) criteria, among treatment-seeking cocaine users, at the Emergency Department or by primary care physicians/Substance Abuse Services in the Florence metropolitan area, Italy.

Inclusion and exclusion criteria and rTMS treatment protocol were reported in our previously published randomized controlled trials (Scarpino, Lanzo et al. 2019, Lolli, Salimova et al. 2021). Stool and saliva samples were obtained from healthy volunteers and CUD patients upon enrollment and immediately frozen at −80°C. For CUD patients, fecal and saliva samples have also been collected and frozen at −80°C after 8 weeks of rTMS treatment. The study protocol was approved by the Azienda Ospedaliero-Universitaria Careggi Ethics Committee (CEAVC SPE. 16.309; MagneTox trial, Jul 17, 2017) and, following the Helsinki Declaration, all patients signed an informed consent form after receiving the study description. Since the Ethics Committee prohibited the withdrawal of pre-existing pharmacological treatments and psychotherapy, psychoactive drugs were either maintained unmodified if chronically administered or titrated/adjusted for steady-state achievement if recently prescribed before starting rTMS.

### Method details

#### Fecal and salivary microbiota characterization

Genomic DNA was extracted using the DNeasy PowerSoil ProKit (Qiagen, Hilden, Germany) from frozen (−80°C) stool and saliva samples, according to the manufacturer’s instructions. Before their processing, saliva samples were centrifuged in a 1.5 mL microcentrifuge tube at 10,000 rpm for 10 min then the supernatants were discarded, and the pellets were collected. Briefly, 0.25 g of stool sample or the salivary pellet were added to a bead beating tube and homogenized with TissueLyser LT (Qiagen, Hilden, Germany) for 5 min at 50 Hz. Afterwards, DNA was captured on a silica membrane in a spin column format, washed and eluted. The quality and quantity of extracted DNA were assessed with both NanoDrop ND-1000 (Thermo Fisher Scientific, Waltham, USA) and Qubit Fluorometer (Thermo Fisher Scientific, Waltham, USA) and then it was frozen at −20°C. Subsequently, total DNA samples were sent to IGA Technology Services (Udine, Italy) where amplicons of the variable V3–V4 region of the bacterial 16S rRNA gene were sequenced in paired-end (2 × 300 cycles) on the Illumina MiSeq platform, according to the Illumina 16S Metagenomic Sequencing Library Preparation protocol. Demultiplexed sequence reads were processed using QIIME2 2021.4.^98^ The sequencing primers and the reads without primers were removed using the Cutadapt tool^99^ while DADA2^100^ was used to perform paired-end end reads merging, filtering and chimeras removal steps after trimming low-quality nucleotides from both forward and reverse reads. This quality trimming has been evaluated separately for saliva and stool samples according to their specific average sequencing quality. Hence, ASVs (amplicon sequence variants) were generated, and the taxonomic assignments have been performed through a scikit-learn multinomial naive Bayes classifier trained on the SILVA database (release 138).^108^^,^^109^ Lastly, PICRUST2 was utilized to predict the expressed METACYC pathways in both environments through the EPA placement algorithm.^101^^,^^110^ Further information about the processing pipeline and the chosen parameters are available in the relative processing Bash script (see data and code availability).

#### SCFAs and MCFAs determination by GC-MS analysis

The qualitative and quantitative evaluation of fecal short chain fatty acids (SCFAs) and medium chain fatty acids (MCFAs) was performed by Agilent gas chromatography-mass spectrometry (GC-MS) system composed with 5971 single quadrupole mass spectrometer, 5890 gas-chromatograph and 7673 autosampler, through our previously described method.^111^ Briefly, just before the analysis, stool samples were thawed and added with sodium bicarbonate 10 mM solution (1:1 w/v) in a 1.5 mL centrifuge tube. Then, the obtained suspensions were sonicated for 5 min, centrifuged at 5000 rpm for 10 min and then the supernatants were collected.

The SCFAs and MCFAs were finally extracted as follows: 50 μL of internal standards mixture, 1 mL of *tert*-butyl methyl ether and 50 μL of HCl 6 M + 0.5 M NaCl solution were added to an aliquot of 100 μL of sample solution (corresponding to 0.1 mg of stool sample) in a 1.5 mL centrifuge tube. Subsequently, each tube was shaken in a vortex apparatus for 2 min and centrifuged at 10,000 rpm for 5 min. Lastly, the solvent layer was transferred to an autosampler vial and processed three times.

Both SCFAs and MCFAs in the samples were analyzed as free acid form using an Agilent J&W DB-FFAP column 30 m length, 0.25 mm internal diameter and 0.25 m of film thickness by using the oven temperatures’ program, as follows: initial temperature of 50 °C for 1 min, then it was increased to 150 °Cat 30 °C/min, finally grow up to 250 °Cat 20 °C/min was held for 6.67 min. A 1 μL aliquot of extracted sample was injected in splitless mode (splitless time 1 min) at 250°C, while the transfer line temperature was 280°C. The used carrier gas was helium and its flow rate was maintained at 1 mL/min for the whole run time. The MS acquisition was carried out in single ion monitoring (SIM) by applying a proper dwell time (20 ms for each ion monitored) to guarantee an acquisition frequency of 4 cycle/s. The quantitative determination of both SCFAs and MCFAs in each sample was carried out by the ratio between the area abundance of the analytes and the area abundance of the respective labeled internal standard (isotopic dilution method). The value of this ratio was named Peak Area Ratio (PAR) and it was used as the abundance of each analyte in the quantitative evaluation. The ionic SCFAs and MCFAs signals and the reference internal standards used for the quantitation of each SCFA and MCFA were reported in Table S7.

#### Transcranial magnetic stimulation (rTMS)

rTMS application was performed as previously described.^44^^,^^45^ Briefly, patients received 15 sessions of high frequency (15 Hz) rTMS with a pulse intensity of 100% (individual threshold levels), 60 pulses per train, an inter-train pause of 15 s, 40 stimulation trains, and a sum of 2,400 pulses in 13 min. The target point of rTMS was the dorsolateral prefrontal cortex and the 5 cm method was employed. A standard figure-of-eight coil MagPro X100 stimulator (MagVenture, Denmark) and active (code MC-F-B65) and sham coils (MCF-P-B65) have been used. The methods for determining the position of the primary motor cortex (M1) area and the motor threshold will be repeated before each rTMS session. The intensity of the rTMS will be determined by mapping the first dorsal interosseous (FDI) muscle response and getting the individual’s resting motor threshold (rMT), which indicates the membrane-related excitability of cortical axons. The rMT will be settled by determining the lowest stimulator output intensity required to obtain 5 out of 10 motors evoked potentials (MEP) greater than 50 μV using the MEP method.^112^ We used a pulse intensity of 100% of their rMT when delivering real rTMS. During the 15 rTMS treatment sessions, the rMT was measured daily for each participant to ensure safety and efficacy. The coil center placed at the left dorsolateral prefrontal cortex was pointing 45° relative to the midsagittal line. The placebo coil had aspect and sound levels identical to the active coil, but the magnetic field was reduced by 80%, although it had a similar cutaneous sensation.^44^

### Quantification and statistical analysis

Statistical analyses on the bacterial communities were performed in R 4.1 with the help of the packages phyloseq 1.36.0^102^, DESeq2 1.32.0^103^ and other packages satisfying their dependencies, as vegan 2.5–7.^104^ Packages ggplot2 3.3.5^105^ and dendextend 1.15.1^106^ were used to plot data and results. Shannon and Pielou’s evenness indices and Observed ASV richness, were used to estimate bacterial diversity in each sample using the function estimate_richness from phyloseq. The evenness index was calculated using the formula E = S/log(R), where S is the Shannon diversity index and R is the Observed ASV richness in the sample; differences in all indices were tested using the Mann-Whitney test.

PCoAs were performed on proportional count data of each sample, adjusted with square root transformation while at the different taxonomic ranks, the differential analysis of abundance was performed with DESeq2 on raw count data. Differential abundances of predicted pathways among healthy controls and cocaine consuming users were determined and displayed using LefSe (linear discriminant analysis effect size) tool^107^on the Galaxy platform.^113^Further information about the statistical analysis regarding the microbiota is available on the relative R Script (see data and code availability). Moreover, the software GraphPad Prism (v.5) was used for the statistical analysis of the fecal SCFAs’ and MCFA’s abundances between healthy controls and CUD patients and among samples collected before and after rTMS treatment. Mann-Whitney test was used to assess differences between unpaired samples while Wilcoxon signed-rank test was used for paired statistical analysis. p-values less than 0,05 were considered statistically significant.

## Data Availability

• All 16S rRNA sequencing data presented in this study are deposited in the NCBI Gene Expression Omnibus (GEO) repository, accession number GSE206765. • This paper does not report original code. • Any additional information required to reanalyze the data reported in this paper is available from the lead contact upon request.
