# Supplementary material for: Oral and fecal microbiota perturbance in cocaine users: Can rTMS-induced cocaine abstinence support eubiosis restoration?
Source: iScience. 2023 Apr 20;26(5):106627. doi: 10.1016/j.isci.2023.106627 (PMC10214473; doi:10.1016/j.isci.2023.106627)
Supplement: Supplementary file 1 — Document S1. Figures S1 and S2 and Tables S1–S7 [file mmc1.pdf]

iScience, Volume *26*

Supplemental information

Oral and fecal microbiota perturbance in cocaine users: Can rTMS-induced cocaine abstinence support eubiosis restoration?

Elisabetta Gerace, Simone Baldi, Maya Salimova, Leandro Di Gloria, Lavinia Curini, Virginia Cimino, Giulia Nannini, Edda Russo, Marco Pallecchi, Matteo Ramazzotti, Gianluca Bartolucci, Brunella Occupati, Cecilia Lanzi, Maenia Scarpino, Giovanni Lanzo, Antonello Grippo, Francesco Lolli, Guido Mannaioni, and Amedeo Amedei

SUPPLEMENTARY FIGURES

Figure S1. ASVs rarefaction curves. (Related to Figures 1, 2 and 3)

(A) ASVs saturation curves for faecal samples of HC and CUD patients.

(B) ASVs saturation curves for saliva samples of HC and CUD patients.

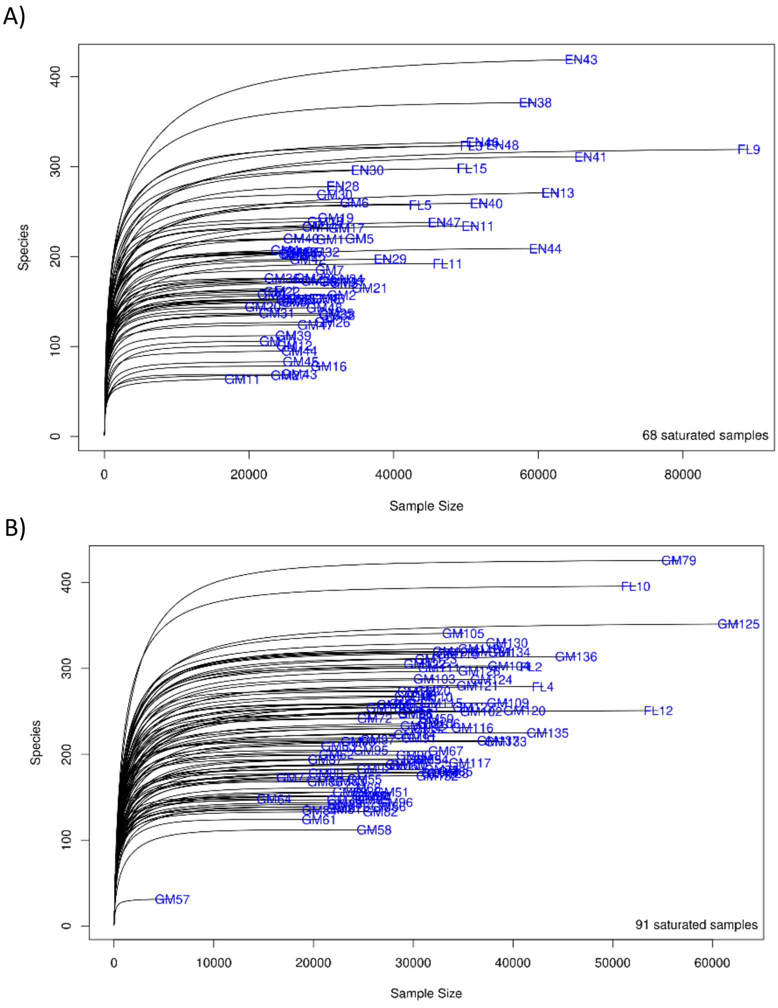

Figure S2. Significant enriched MetaCyc pathways among HC and CUD patients. (Related to Figure 1)

(A) Significant enriched MetaCyc pathways in faecal samples of HC and CUD patients.

(B) Significant enriched MetaCyc pathways in saliva samples of HC and CUD patients.

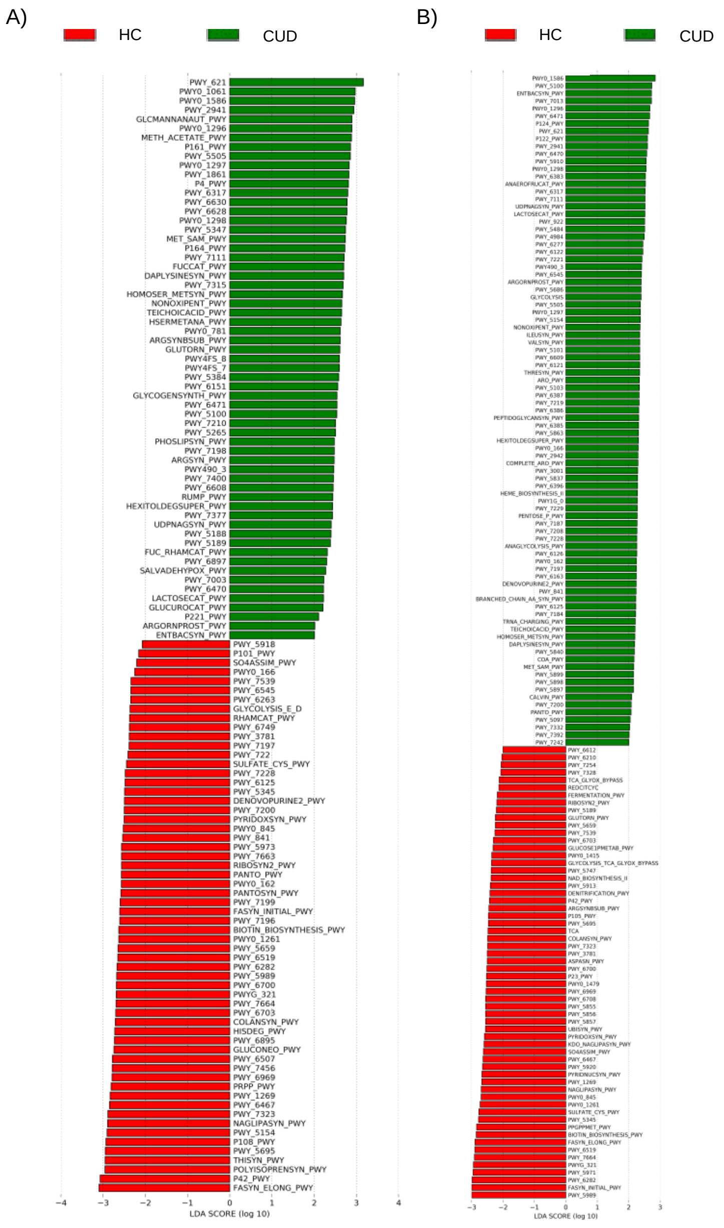

SUPPLEMENTARY TABLES

Table S1. Clinical features of enrolled CUD patients. (Related to STAR Methods)

Features	CUD patients	HC
Sex, %	Male: 83%<br>Female: 17%	Male: 85%<br>Female: 15%
Age at enrolment, mean (SD)	40.4 (8.9)	43.8 (7.9)
Time of cocaine use (years), %	<5 years: 19%<br>5-10 years: 16%<br>>10 years: 62%<br>NR: 3%	-
Frequency of cocaine consumption, %	<= 4/week: 51%<br>1/day:29%<br>>1/day: 14%<br>NR: 6%	-
Modality of assumption, %	Single 84% (Sniffing:67%, Smoking:37%, intravenous:16%)<br>Multiple: 16%	-
Co-dependence	Yes: 10%<br>No: 8%<br>NR: 83%	-
Alcohol	Yes: 67%<br>No: 8%<br>NR: 25%<br>Urine+:62%	-
THC	Yes:35%<br>No: 25%<br>NR:40%<br>Urine+: 27%	-
Opioids	Yes: 5%<br>No: 25%<br>NR: 40%<br>Urine+:5%	-
Methadone	Yes: 5%<br>No: 10%<br>NR:70%	-
Amphetamine	Yes: 2%<br>No: 10%<br>NR:88%<br>Urine+:41%	-
Tobacco	Yes:76%<br>No:3%<br>NR: 21%	Yes:45%<br>No: 55%-
Benzodiazepine	Yes: 0<br>No: 14%<br>NR:86%	-

Drugs (antidepressants, stabilizers, antipsychotics, atomoxetine, disulfiram, and buprenorphine)	Yes: 52%<br>No: 48%	-

Table S2. PERMANOVA tests at all taxonomic ranks between faecal and saliva samples of HC and CUD patients. (Related to Figure 1)

Faecal samples					
**Rank**	**SumsOfSqs**	**MeanSqs**	**F.Model**	**R2**	**P value**
Phyla	0.24474	0.24474	11.4815	0.175339	< 0.0001
Classes	0.50746	0.50746	10.9672	0.168812	< 0.0001
Orders	0.27520	0.27520	0.0671	0.157133	< 0.0001
Families	0.60432	0.60432	8.5252	0.136349	< 0.0001
Genera	0.78023	0.78023	6.4557	0.106784	< 0.0001
**Saliva samples**					
**Rank**	**SumsOfSqs**	**MeanSqs**	**F.Model**	**R2**	**P value**
Phyla	0.19775	0.09887	9.0607	0.170761	< 0.0001
Classes	0.26907	0.13454	5.5295	0.111641	< 0.0001
Orders	0.18869	0.09435	6.5598	0.129743	< 0.0001
Families	0.30488	0.15244	4.5579	0.093866	< 0.0001
Genera	0.37763	0.18881	3.8818	0.081070	< 0.0001

Table S3. Summary of the taxonomic analysis of the obtained ASVs from faecal and saliva samples of CUD patients and HC. (Related to Figure 2)

	**Faecal samples**		
**Rank**	**Count**	**Assigned ASV**	**% ASV**
Phylum	14	13	92,85
Class	24	23	95.83
Order	58	56	96.55
Family	108	101	93.51
Genus	312	290	92.94
	**Saliva samples**		
**Rank**	**Count**	**Assigned ASV**	**% ASV**
Phylum	17	14	82.35
Class	27	24	88.88
Order	62	57	91.93
Family	108	99	91.66
Genus	254	233	91.73

Table S4. Significant differentially abundant taxa in faecal samples of CUD patients compared to HC. (Related to Figure 3A)

Log2FoldChange	padj	Regulation		
3.546	0.031	*down*	Christensenellaceae	**Family**
5.162	4.02e-5	*down*	Desulfovibrionaceae	
-2.389	5.3e-4	up	Erysipelotrichaceae	
1.941	0.013	*down*	Lachnospiraceae	
-3.005	0.035	*up*	*[Clostridium]_innocuum_group*	**Genus**
2.275	0.016	*down*	*[Eubacterium]_ventriosum_group*	
3.737	0.031	*down*	*[Eubacterium]_xylanophilum_group*	
-1.362	0.016	*up*	*[Ruminococcus]_torques_group*	
2,79088	4.02e-5	*down*	*Alistipes*	
1,970077	1.4e-4	*down*	*Bacteroides*	
2,674471	0.026	*down*	*Barnesiella*	
-1,54938	6.52e-5	*Up*	*Blautia*	
-2,0095	0.025	*up*	*Clostridium_sensu_stricto*	
-1,16503	0.026	*up*	*Collinsella*	
5,746095	2.96e-4	*down*	*Coprobacter*	
-1,26536	0.033	*up*	*Dorea*	
-4,25861	4.02e-5	*up*	*Escherichia-Shigella*	
-6,39956	0.034	*up*	*Eubacterium*	
4,333503	0.004	*down*	*GCA-900066575*	
-5,4812	0.001	*up*	*Holdemanella*	
-9,40247	1.65e-4	*up*	*Megamonas*	
2,545247	0.008	*down*	*Odoribacter*	
5,873049	0.048	*down*	*Oscillospira*	
3,030045	0.039	*down*	*Paraprevotella*	
3,395048	0.013	*down*	*Parasutterella*	
-6,6863	9.68e-4	*up*	*Peptococcus*	
5,403781	0.015	*down*	*Prevotellaceae_NK3B31_group*	
-1,93903	0.004	*up*	*Romboutsia*	
-3,88444	0.015	*up*	*Rothia*	
-3,16199	0.007	*up*	*Senegalimassilia*	
-2,509	9.48e5	*up*	*Streptococcus*	
3.641	0.018	*down*	*Sutterella*	
-2.526	0.015	*up*	*Turicibacter*	
3.107	0.002	*down*	*UCG-002*	

Table S5. Significant differentially abundant genera in saliva samples of CUD patients compared to HC. (Related to Figure 3B)

Log2FoldChange	padj	Regulation		
-3.578	3.33e-4	*up*	*[Eubacterium]_saphenum_group*	
1.83	0.024	*down*	*Aggregatibacter*	
2.466	1.63e-9	*down*	*Alloprevotella*	
-6.033	1.88e-6	*up*	*Alloscardovia*	
1.413	0.004	*down*	*Capnocytophaga*	
1.186	0.028	*down*	*Harmophilus*	
2.090	0.025	*down*	*Johnsonella*	
1.147	0.017	*down*	*Lachnoanaerobaculum*	
-2.743	5.54e-4	*up*	*Lactobacillus*	
2.206	9.22e-4	*down*	*Neisseria*	**Genus**
-2.414	0.024	*up*	*Olsenella*	
1.676	0.003	*down*	*Peptostreptococcus*	
1.284	5.69e-4	*down*	*Porphyromonas*	
-6.065	3.34e-4	*up*	Prevotellaceae_UCG-001	
-2.162	0.020	*up*	*Rikenellaceae_RC9_gut_group*	
-1.066	0.004	*up*	*Rothia*	
-4.510	0.031	*up*	*Staphylococcus*	
-1.551	0.012	*up*	*Treponema*	

Table S6. Faecal SCFAs and MCFAS abundances of HC and CUD patients. (Related to Figure 4)

**Percentage of each faecal SCFA and MCFA** (median (IQR); %)	**CUD**	**HC**	**p value**
Acetic acid	54.54 (10.69)	53.40 (9.34)	0.508
Propionic acid	20.49 (10.80)	17.33 (3.48)	0.106
Butyric acid	11.38 (7.12)	12.04 (7.88)	1.0e-4
isoButyric acid	2.02 (2.23)	1.93 (1.66)	0.963
isoValeric acid	1.89 (2.04)	1.43 (1.36)	0.550
2-MethylButyric acid	1.88 (2.26)	1.45 (1.54)	0.730
Valeric acid	3.27 (1.59)	2.91 (1.05)	0.430
Isohexanoic acid	0.65 (0.21)	0.04 (0.04)	0.014
Hexanoic acid	0.64 (0.10)	0.94 (1.39)	1.0e-4
Heptanoic	1.05 (0.83)	0.11 (0.16)	0.028
Octanoic	0.60 (0.12)	0.01 (0.01)	0.106
Nonanoic	0.52 (0.01)	0.01 (0.01)	0.019
Decanoic	0.52 (0.01)	0.00 (0.01)	0.015
Dodecanoic	0.55 (0.02)	0.01 (0.02)	0.019

Table S7. Retention time, quali- and quantitative ion and their internal standard for each analyte. (Related to Figure 4)

	Rt (min.)	Quant. Ion *(m/z)*	Qual. Ion *(m/z)*	ISTD [Quant. Ion *(m/z)*]
**SCFAs (%)**				
Acetic Acid	5.65	60	-	[$^{2}H_{3}$]Acetic [63]
Propionic Acid	5.050	74	73	[$^{2}H_{5}$]Propionic Acid [77]
Iso-Butyric Acid	5.171	73	88	[$^{2}H_{7}$]iso-Butyric Acid [77]
Butyric Acid	5.459	60	73	[$^{2}H_{5}$]Propionic Acid [77]
Iso-Valeric Acid	5.649	60	73	[$^{2}H_{9}$]iso-Valeric Acid [63]
2-Methylbutyric Acid	5.649	74	73	[$^{2}H_{9}$]iso-Valeric Acid [63]
Valeric Acid	5.972	60	73	[$^{2}H_{9}$]Valeric Acid [63]
**MCFAs (%)**				
Iso-Hexanoic Acid	6.276	60	73	[$^{2}H_{11}$]Hexanoic Acid [63]
Hexanoic Acid	6.462	60	73	[$^{2}H_{11}$]Hexanoic Acid [63]
Heptanoic Acid	6.952	60	73	[$^{2}H_{11}$]Hexanoic Acid [63]
Octanoic Acid	7.442	60	73	[$^{2}H_{13}$]Octanoic Acid [63]
Nonanoic Acid	7.920	60	73	[$^{2}H_{13}$]Octanoic Acid [63]
Decanoic Acid	8.387	60	73	[$^{2}H_{13}$]Octanoic Acid [63]
Dodecanoic Acid	9.286	60	73	[$^{2}H_{13}$]Octanoic Acid [63]
